# Improved Optimization of a Coextrusion Die with a Complex Geometry Using the Coupling Inverse Design Method

**DOI:** 10.3390/polym15153310

**Published:** 2023-08-04

**Authors:** Xinyu Hao, Guangdong Zhang, Tong Deng

**Affiliations:** 1 School of Mechanical Engineering, Yancheng Institute of Technology, Yancheng 224051, China; haoxinyu@ycit.edu.cn; 2 The Wolfson Centre for Bulk Solids Handling Technology, Faculty of Engineering and Science, University of Greenwich, Chatham ME4 4TB, UK; t.deng@greenwich.ac.uk

**Keywords:** coextrusion, optimization design, inverse design, coupling method, thermoplastic polyurethane, barium sulfate

## Abstract

The main challenge in a polymer coextrusion process is to have a good die design prior to the process, which can minimize the geometric errors that are caused by extrusion swell and interface motion. For this purpose, a coupling method of optimization and inverse design for a coextrusion die was studied for a medical striped catheter. In the study, the main material was thermoplastic polyurethane (TPU), and the auxiliary material was TPU filled with 30 wt% barium sulfate. An overall optimization design method was used to optimize the geometry of the extrusion die channel for the striped catheter, which had a complex geometry. In the global optimization process, the local inverse design method was used to design the inlet of the auxiliary material. The non-linear programming by quadratic Lagrangian (NLPQL) algorithm was used to obtain the optimal geometric solution of the coextrusion die runner. The experimental verification results showed that the coupling method for coextrusion die design improved the design efficiency of the coextrusion die remarkably. The value of the objective function, which was used to measure the geometric error of the product, was reduced by 72.3% compared with the initial die design.

## 1. Introduction

The polymer coextrusion process [1] is a type of extrusion process that can be used to manufacture polymer composites with different structures, complicated functional areas, or multiple colors. Coextrusion technology is widely used for composite films [2], sheets, pipes [3], and profiles [4]. For medical products, the coextrusion is used to produce single-lumen or multi-lumen catheters [5], such as balloon dilatation catheters, stent implantation catheters, urinary catheters, and hemodialysis catheters. As minimally invasive surgery advances, the need for catheters with smaller dimensions and greater precision has become more prevalent [6]. However, in the coextrusion process, the coupling problem of polymer die swell and coextrusion interface movement [7] seriously restricts the accuracy of the catheter geometry. This development poses new challenges to the design and manufacturing of coextrusion dies. As the flow channel size of the coextrusion die approaches the millimeter scale and the dimensional accuracy of the extrudate reaches the micron level [8,9], concerns regarding extrusion deformation and dimensional control in the polymer coextrusion process have become more pronounced.

The traditional approach to design coextrusion dies relies typically on a trial-and-error methodology [10,11]. This approach involves iterative adjustments and practical experimentation to optimize the die design. However, the trial-and-error approach suffers from several limitations that hinder its effectiveness. These limitations include time-consuming processes, high costs, limited exploration of design alternatives, subjective decision making relying on intuition, limited insights into the flow behavior, and difficulties in quantifying the performance.

In recent years, the development in numerical simulation technologies of polymer extrusion [12] has provided numerical solutions to enhance the operational efficiency, minimize the costs, and shorten the production cycles during the design and manufacturing of coextrusion dies. The numerical simulation allows for an in-depth flow analysis of the individual polymer materials, providing insights into their rheological behavior during extrusion. The analysis enables an accurate prediction of the die swell [13] and interface offset [14]. Additionally, this analysis aids in identifying potential concerns, such as flow imbalance, interfacial defects [15], or uneven die swell, which can impact layer uniformity and the overall quality of the end product [16]. Consequently, it facilitates the optimization of flow channel geometry and process parameters within the coextrusion die, ensuring the attainment of the desired geometric accuracy for the extrudate.

Based on the analysis and simulation results, the die geometry [17], flow channels [18], manifold configuration [19], and other components can be optimized to achieve the desired performance criteria, such as layer distribution, layer thickness control [16], interfacial quality, and shape of the extrudate [20,21]. The design variables involve adjusting dimensions, angles, land lengths, and lip designs to control the material flow and achieve a uniform layer distribution. Optimization design methods include mainly the response surface method [18], gradient-based method [22], sensitivity analysis [23], artificial neural networks [24], and genetic algorithm [25].

The existing coextrusion die designs commonly take the uniform layer distribution within the die as the design target. In the existing numerical simulations, the layer distribution within the die was considered, but it did not include the die swell and interface offset outside the die. For micro coextrusion, in the optimization design of the coextrusion dies aiming for geometric accuracy of the extrudate [21], both the die swell and interface offset are essential and must be considered. Due to the existence of a free surface and moving interfaces, the numerical solutions of the design point may be non-convergent, which leads to the non-convergence of the optimal design. Additionally, the existing commercial software, namely ANSYS Polyflow, does not support the inverse design method for more than two free surfaces [26,27], so the application of the inverse design with a free surface and moving interface for the coextrusion die can be implemented using any commercial software directly.

The extrusion swell and the interface motion raise challenges in achieving the optimal coextrusion die design using the traditional trial-and-error methods. However, with the advantages in numerical simulation and optimization technologies, it has become feasible to design coextrusion dies with high precision, efficiency, and cost-effectiveness. This study introduces a new coupling design method for coextrusion dies with complex geometries, aiming to enhance the geometric accuracy in the final coextrusion products. As a practical example, a medical striped catheter was utilized for the coextrusion die design. The optimization design successfully identified the optimal solution for the coextrusion die, which was subsequently validated experimentally.

## 2. Problem Definitions for the Coextrusion

**Figure 1:**
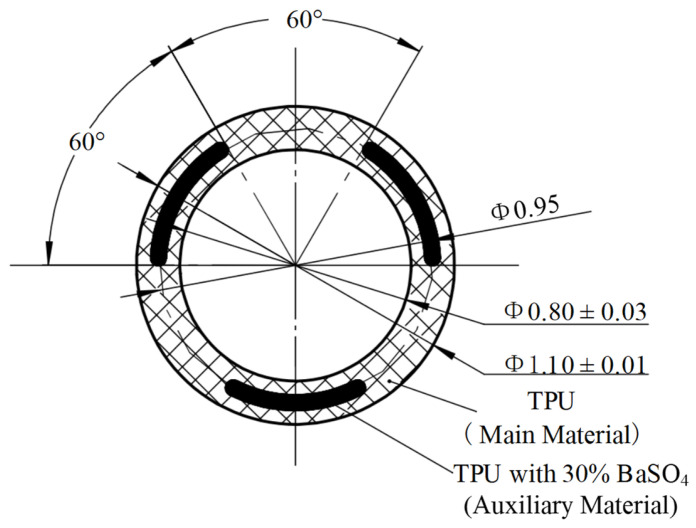
Coextrusion product geometry (Unit: mm).

In this study, the coextruded product studied was a striped single-lumen catheter, as shown in Figure 1 [6,28], which had an external diameter of 1.10 ± 0.01 mm, an inner diameter of 0.80 mm ± 0.03 mm, and a wall thickness of 0.15 mm. The main material used in this study was thermoplastic polyurethane (TPU, Lubrizol TPU2363-65D [29]), which was specially designed for medical material applications. The TPU2363 series has exceptional hydrolytic stability across various durometers and offers favorable mechanical and chemical resistance properties. The auxiliary material was TPU modified with barium sulfate (BaSO_4_), in which the TPU2363-65D was filled with 30 wt% BaSO_4_ as the X-ray contrast agent, also known as the barium line. The thickness of the barium wires was 0.05 mm, and they were uniformly distributed along a circle of Φ = 0.95 mm, with an angular interval of 60°.

**Figure 2:**
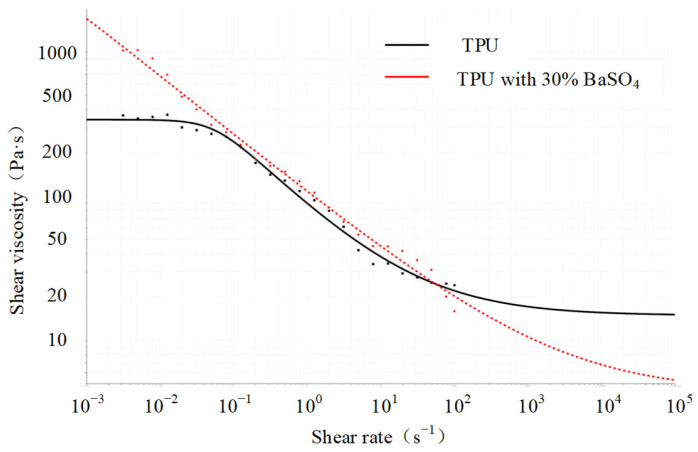
Viscosity–shear rate curve (TPU and TPU with 30 wt% BaSO_4_).

There are significant differences [30] in the rheological and processability of TPU filled with BaSO_4_ and TPU without BaSO_4_. The shear rate–viscosity curve of a polymer melt is essential, especially the slope of the curve or its power law exponent. If the viscosities of different polymers do not match, it may cause the offset of the position of the coextrusion interface and affect the stability of the interface. The rheological properties of the TPU and the modified TPU were measured by HR-2 rheometer (TA Instruments, Discovery, USA) equipped with a parallel-plate geometry (25 mm diameter) [31]. Constant temperature frequency scanning was carried out on the two materials. The scanning temperature was 220 °C, and the scanning frequency range was 0.1–100 rad/s. The curves of the shear viscosity of the two materials with the shear rate were obtained, as shown in Figure 2. Since the barium sulfate modified TPU was filled with 30 wt% BaSO_4_, its rheological properties differed from the (non-modified) TPU. When the shear rate was lower than 0.1 s^−1^, the shear viscosity of the modified TPU was much higher than that of the TPU; when the shear rate was between 0.1 s^−1^ and 77 s^−1^, the shear viscosity of the modified TPU was slightly higher than that of the TPU. When the shear rate was higher than 77 s^−1^, the shear viscosity of the modified TPU was smaller than that of the TPU.

The shear rate–viscosity curves of the two materials aligned with the rheological curve characteristics of typical shear-thinning fluids, and the shear-rate-dependent constitutive model of viscosity, namely the Bird–Carreau pure viscous model, and its rheological parameters are shown in Table 1.

## 3. Finite Element Model of the Coextrusion

The coextrusion finite element model was established utilizing the commercial software ANSYS Polyflow (ver. 2022 R1) [12], which was extensively utilized for the simulation and analysis of the flow behavior, layer distribution, die swell, and interface movement. In the coextrusion process, it is commonly assumed that the two polymer melts behave as generalized Newtonian fluids and flow under isothermal conditions [32]. This assumption is based on the understanding that polymer melt flow tends to exhibit higher viscosity and lower Reynolds numbers, thus lending itself to a laminar flow regime [15]. Furthermore, the fluid is typically treated as incompressible [33], resulting in a constant fluid density throughout the process. Additionally, it is often justifiable to neglect the influence of gravity and inertial forces [33] on the coextrusion die process, as they are relatively small when compared with the dominant viscous forces exerted by the melt.

ANSYS Polyflow supports a wide range of material models commonly used in polymer processing simulations, including the Bird–Carreau pure viscosity model, the differential viscosity model PTT [7], and the K-BKZ Model [34]. In this study, the Bird–Carreau model was adopted to describe the viscosity behavior of non-Newtonian fluids, considering their shear rate dependence. The choice of this model was justified by the material’s insignificant elastic properties.

**Figure 3:**
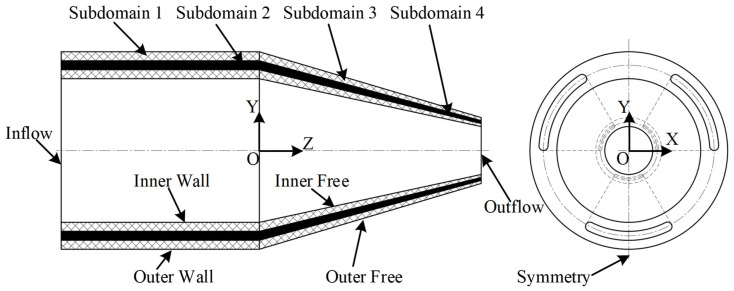
Definition of geometric model and boundary sets in the coextrusion process.

A geometric coextrusion model was developed, as shown in Figure 3. Since the geometric model was symmetrical about the Y-Z plane, the symmetric boundary conditions served to simplify the calculation, so the simulation model adopted only half of the geometry in the computation domain.

Along the Z-axis to the right is the die land (within the die) and the free jet (outside the die). The die land length was preset to 22 mm, and the free jet length to 20 mm. The geometric modeling was implemented in ANSYS DesignModeler software.

In the coextrusion numerical simulation of ANSYS Polyflow, in addition to the inlet (inflow), outlet (outflow), wall (inner wall, outer wall), and free surface (inner free, outer free) boundary conditions shown in Figure 3, it was also necessary to define the moving interface boundary conditions. The computational domain and boundary conditions were defined as follows:

➢ Main Material: Subdomain 1 (die land), Subdomain 3 (free jet).
➢ Auxiliary Material: Subdomain 2 (die land), Subdomain 4 (free jet).
➢ Inflow: Inlet flow rate of the main and auxiliary materials was 35.84 mm^3^/s and 7 mm^3^/s, respectively.
➢ Outflow: Take-up velocity was 160 mm/s.
➢ Inner and Outer Walls: No-slip boundary condition was applied.
➢ Inner and Outer Free Surfaces: Position of the free surface was unknown.
➢ Symmetry: Symmetry plane.
➢ Moving Interface: Interface between the main and auxiliary material.

**Figure 4:**
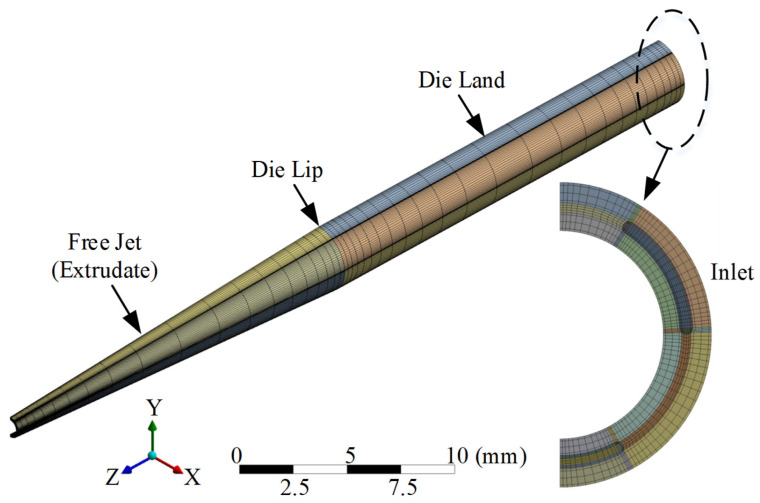
Mesh model (Die Lip and Free Jet).

The finite element model adopted a hexahedral structured mesh model, as shown in Figure 4. The number of elements was 72,000, the number of nodes was 78,591, and the output unit was set to mm. The mesh generation method adopted the overall sweep method [35], which was used to create a mesh for structures with geometric features that could be extruded along a path. The number of meshes in the extrusion direction of the die land and free jet was 25 and 20, respectively, and a local mesh control method [36] with the bias types of the sweep method was applied at the die lip. The sweep bias type was the grading of the elements towards both ends. The sweep bias factor of the die land and free jet was 10 and 9, respectively.

## 4. Draw Ratio and Die Land Length

At the beginning of the design of a catheter coextrusion die, it is first necessary to select the reasonable size of the draw ratio. The main reason is that the overall size of the coextrusion die needs to be determined according to the draw ratio. The draw ratio describes how stretched is the catheter after it leaves the die exit. In catheter extrusion, the definition of the draw ratio [37,38] includes the diameter draw ratio (*DDR*), wall draw ratio (*WDR*), area draw ratio (*ADR*), draw ratio balance (*DRB*), and sizing ratio (*SR*). Usually, the draw ratio (*DR*) refers to the area draw ratio (*ADR*). If the draw ratio is too large, the molecular orientation effect of the polymer will be significant, and the extruded part will be broken. If the draw ratio is too small, the molecular orientation of the polymer will be insufficient, causing a melt fracture within the die.

**Figure 5:**
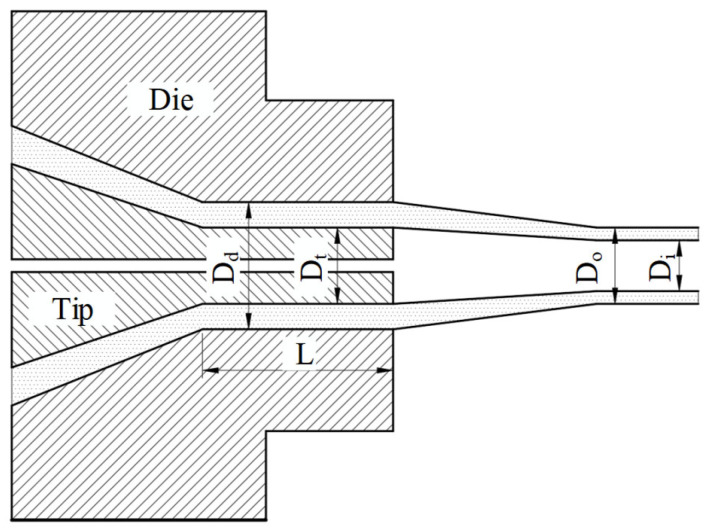
Definition of Geometric Variables in Draw Ratio.

The diameter draw ratio (*DDR*), the ratio of the average of the inner and outer diameters of the die to the average of the inner and outer diameters of the catheter, is defined as follows:

(1) $$DDR=\left(D_{d}+D_{t}\right)/\left(D_{o}+D_{i}\right)$$

where $D_{d}$ is the inner diameter of the flow channel of the die land, $D_{t}$ is the outer diameter of the end of the mandrel, $D_{o}$ is the outer diameter of the catheter, $D_{i}$ is the inner diameter of the catheter, and *L* is the die land length, as shown in Figure 5.

The wall draw ratio (*WDR*) is the ratio of the wall thickness of the die slit to the catheter and is defined as follows:

(2) $$WDR=\left(D_{d}-D_{t}\right)/\left(D_{o}-D_{i}\right)$$

The thickness (*H*) of the die slit is half the difference between the outer diameter of the end of the mandrel in the die land and the inner diameter of the die runner.

(3) $$H=\left(D_{d}-D_{t}\right)/2$$

From Equations (2) and (3),

(4) $$H=WDR\times \left(D_{o}-D_{i}\right)/2$$

The area draw ratio (*ADR*) is the ratio of the cross-sectional area of the die exit to the cross-sectional area of the catheter and is defined as follows:

(5) $$ADR=(D_{d}^{2}-D_{t}^{2})/\left(D_{o}^{2}-D_{i}^{2}\right)$$

For incompressible fluids, the area draw ratio can also be defined as the ratio of the take-up velocity to the extrusion velocity at the die exit.

(6) $$ADR=\upsilon_{draw}/\upsilon_{exit}$$

From Equations (1), (2) and (5), it can be seen that the area draw ratio can also be defined as the product of the diameter draw ratio (*DDR*) and the wall draw ratio (*WDR*):

(7) ADR=DDR×WDR

The draw ratio affects not only the die design but also the pressure drops of the coextrusion die. Generally, the pressure drop should be within the allowable range. If the pressure drop (pressure loss) is too significant, it may cause forming difficulties. When the inlet volume flow rate is constant, the more complex the die geometry, the smaller the thickness (*H*) of the die slit and the greater the pressure drop of the coextrusion die. As the draw ratio increases, it can be seen from Equation (4) that the thickness (*H*) of the die slit increases accordingly. According to the slit die theory, the pressure drop of the die decreases accordingly, and the manufacturing cost of the coextrusion die also decreases accordingly. However, too high a draw-to-draw ratio can lead to excessive molecular orientation effects of the polymer and breakage of the extruded part. According to the material manual, the recommended draw ratio (*DR*) of TPU2363-65D ranges from 1 to 20.

**Figure 6:**
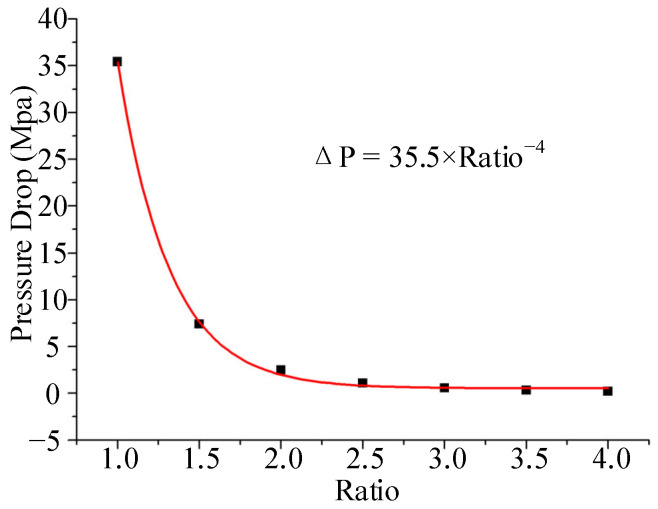
Relationship between pressure drop and size magnification.

It can be seen from the above discussion that the selection of the draw ratio depends mainly on the pressure drop of the coextrusion die and the material properties. In order to obtain the relationship curve between the pressure drop of the coextrusion die and the draw ratio, the cross-sectional size of the die is enlarged proportionally relative to the cross-sectional size of the coextruded product shown in Figure 1, and the size magnification ratio is designated as the Ratio (the value range is 1–4). From Equations (1), (2) and (5), it can be seen that the diameter draw ratio (*DDR*) and the wall draw ratio (*WDR*) are equal to the Ratio, and the area draw ratio (*ADR*) is equal to the Ratio^2^. The die land length of the extrusion die was tentatively determined to be 22 mm based on experience. The coextrusion numerical simulation (including only the die land) resulted in the relationship curve between the pressure drop and the size magnification ratio, as shown in Figure 6.

The relationship between the pressure drop and the size magnification ratio was obtained by fitting ΔP = 35.5 × Ratio^−4^, and the correction coefficient of determination (Adj. R-Square) was 0.9997. When the size magnification ratio was 1, that is, when the cross-sectional size of the die was the same as that of the coextruded product, the pressure drop in the die land of the coextruded die was 35.4 MPa, which was far beyond the processing capacity of the screw extruder. When the size enlargement ratio was 3, the pressure drop in the die land was 0.56 MPa. When the size enlargement ratio was greater than 3, the change in pressure drop was negligible. In summary, the magnification ratio (Ratio) of the cross-sectional size relative to the cross-sectional size of the extrudate was 3. That is, the diameter draw ratio (*DDR*) was 3, the wall draw ratio (*WDR*) was 3, and the area draw ratio (*ADR*) was 9. It can be seen from Figure 1 that the outer diameter of the coextruded catheter was 1.1 mm, and the inner diameter was 0.8 mm. According to the magnification ratio, the inner diameter of the die runner in the die land was 3.3 mm, the outer diameter of the end of the mandrel was 2.4 mm, and the thickness of the die slit was 0.45 mm. The traction speed was 160 mm/s, so according to Equation (6), the extrusion speed was 17.8 mm/s.

For catheter coextrusion, in addition to the inner diameter of the die runner in the die land and the outer diameter of the mandrel, the selection of the die land length is essential. According to the slit die theory [39], the pressure drop in the die land is proportional to the die land length. A reasonable length is beneficial to reduce the extrusion swell and stabilize the size of the extruded part. However, because the die land usually has the smallest wall thickness, it has the most significant flow resistance, so the length of the die land will lead to a significant extrusion pressure drop. In addition, the excessive length will cause the end of the mandrel to bend under compression, especially for a mandrel with a small diameter. The ratio of the length of the die land (*L*) to thickness (*H*) of the die slit is usually between 20 and 50 [39], so the length is between 9 mm and 22.5 mm. For the coextrusion process, the length of the die land must be large enough to ensure the bonding strength of the material junction; therefore, the length was still selected as 22 mm in our study.

## 5. An Improved Coextrusion Die Design Method

Compared with the traditional extrusion die design [40,41,42], the coextrusion die design is more complicated due to the coupling of the free surface and the coextrusion moving interface and the difference in the rheological properties of the materials. In the coextrusion process, the mechanism of the interfacial movement is relatively complicated, and it is generally believed that the existence of the pressure difference on the interface causes the interfacial movement. Due to the coupling effect of the extrusion swell and interface motion, the decoupling calculation cannot be realized using the optimization or inverse design method alone, making the design more complex. Thus, this paper proposes a coupling method for a coextrusion die design with the geometric accuracy of the coextrusion products as the design goal.

The coextrusion die design coupling method combines global optimization and the local inverse method to optimize the inlet geometry of the die land of the main and auxiliary materials, respectively. This method aimed to achieve optimal geometric accuracy of the striped catheter. The coextrusion numerical simulation framework consisted of five functional modules, namely the pre-processing module (ANSYS DesignModeler for geometric modeling, ANSYS Meshing for meshing), the solver module (ANSYS Polyflow), the post-processing module (ANSYS CFD-Post), optimization design modules, and parameter sets. The arbitrary Lagrangian–Eulerian (ALE) method [43] in ANSYS Polyflow was employed to handle large deformations of the mesh and track the movement of the materials during the coextrusion process. It allowed the mesh to move and adapt to the flow conditions, ensuring an accurate representation of the material interfaces and their deformation.

**Figure 7:**
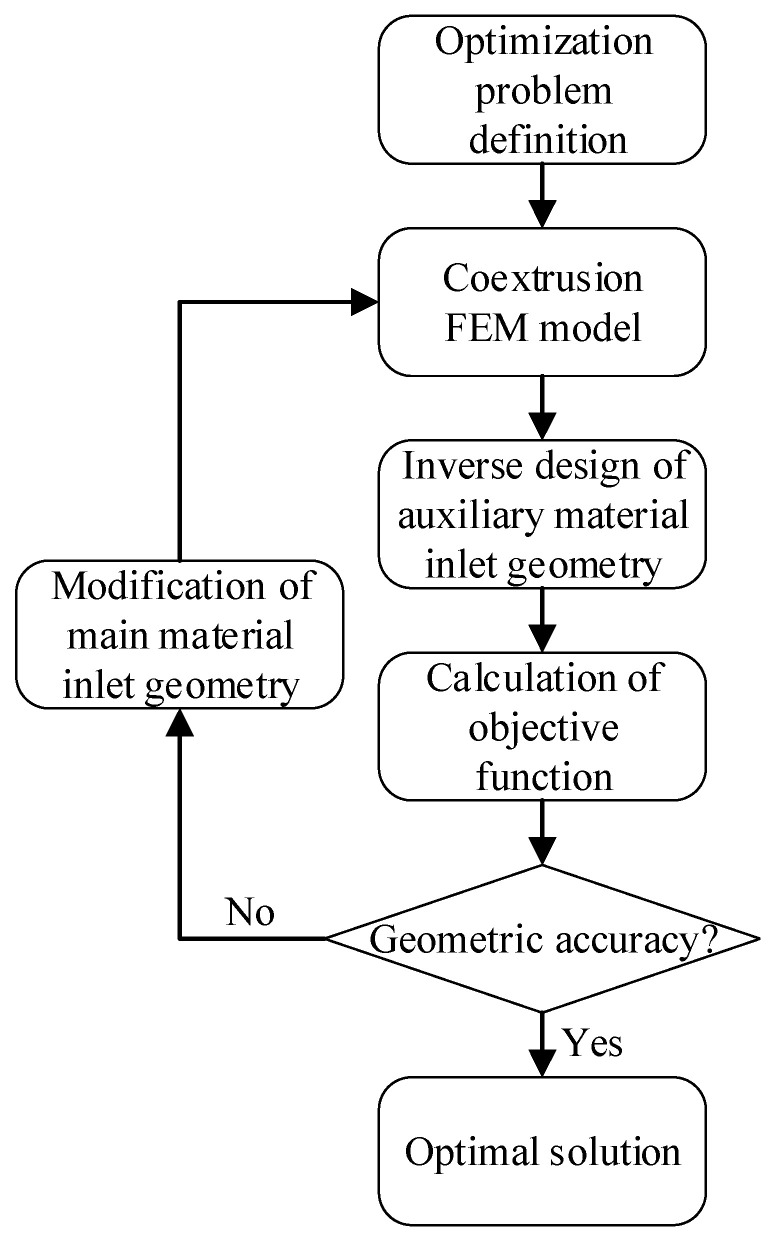
Design process of coextrusion die.

The overall optimization design method was used to optimize the geometry of the die land of the coextrusion die, as shown in Figure 7. The optimization design goal was the geometric accuracy of the main material. In the optimization design process, the interface position of the coextrusion material (the initial geometry of the outlet of the free jet of the auxiliary material) was taken as the design goal, and the inlet geometry of the die land of the auxiliary material was reversely designed by using a local inverse design method. The optimization solver judged whether it converged according to the optimization iteration situation. If it converged, the optimal solution was obtained. If it did not converge, the optimization solver corrected the geometric parameters of the main material inlet until the optimization process converged.

The optimization design of the coextrusion die was aimed at the geometric accuracy of the main material. It was specifically constructed based on the principle of minimizing the displacement of the reference point, which was to minimize the displacement of the target point of the extrudate. The sum of the squares of the Euclidean distance between the target point on the coextruded product and the corresponding actual point after extrusion was the objective function of the optimization design. The expected value of the objective function was 0, as shown below.

(8) $$F_{obj}=\overset{n}{\underset{i=1}{\sum }}{\left(\left(q_{i}-p_{i}\right)/∆x_{i}^{0}\right)}^{2}$$

where *F_obj_* is the objective function, i is the serial number of the target point, n is the number of target points, *q_i_* is the *i*-th target point, *p_i_* is the actual point after extrusion corresponding to the *i*-th target point, and ∆*x_i_* is the design tolerance value of the *i*-th dimension size.

**Figure 8:**
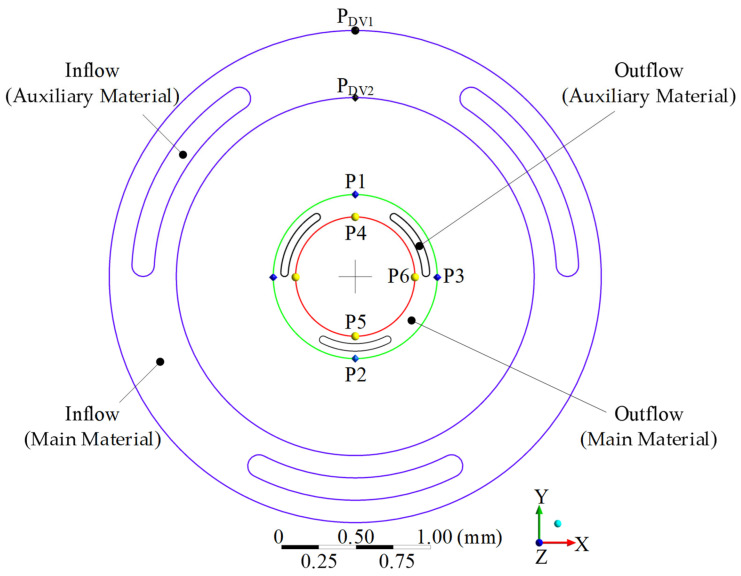
Initial inlet of die land and outlet of the extrudate.

The target points of the optimization design were the points P1~P3 on the outer circle (Green) and the P4~P6 on the inner circle (Red) at the cross section of the main material of the coextruded product, as shown in Figure 8. The target dimensions and tolerances of the outer and inner circles on the main material were 1.1 ± 0.01 mm and 0.8 ± 0.03 mm, respectively. Since the inlet geometry of the auxiliary material was designed by the inverse design method, the geometry of the auxiliary material on the extruded part was consistent with the target geometry, and it did not need to be used to calculate the objective function in the optimization design.

The inner diameter of the die runner was 3.3 mm, the outer diameter of the end of the mandrel was 2.4 mm, and the length of the die land and the free jet was 22 mm and 20 mm, respectively. In the coextrusion die design, the design variables used in the global optimization design were the inner diameter (DV1) of the die land runner (see P_DV1_) and the outer diameter (DV2) of the mandrel end (see P_DV2_), as shown in Figure 8.

The design variables (DV1 and DV2) and their value ranges are described in Table 2.

**Figure 9:**
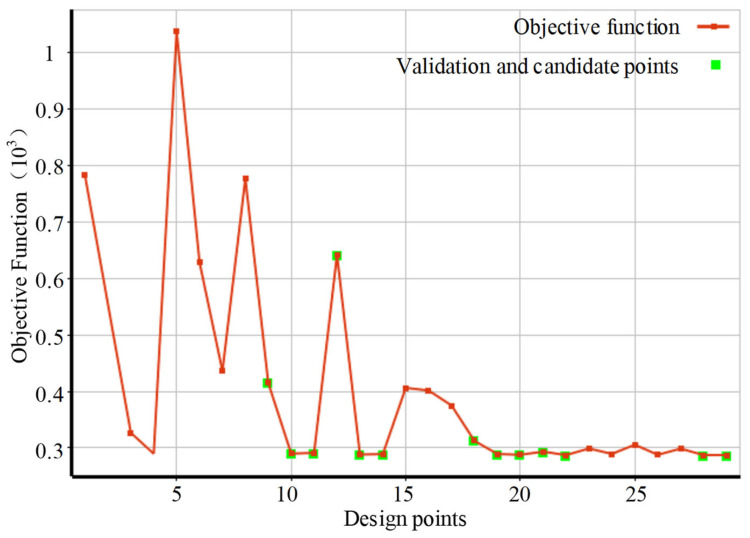
Optimization design process.

Figure 9 describes the changing trend of the objective function in the optimization design process. The adaptive single-objective optimization method based on gradient optimization and the non-linear programming by quadratic Lagrangian (NLPQL) algorithm were adopted, and the total number of design points was 29. There were 29 CFD analyses carried out by Polyflow, and each single CFD analysis required approximately 1 h. During the optimization process, the objective function was reduced from 1036.5 to 287.35, which was a reduction of 72.3%. The results show that the optimum was reached after 19 design points, while the remaining design points were used to build confidence in the numerical solution.

## 6. Results and Discussion

**Figure 10:**
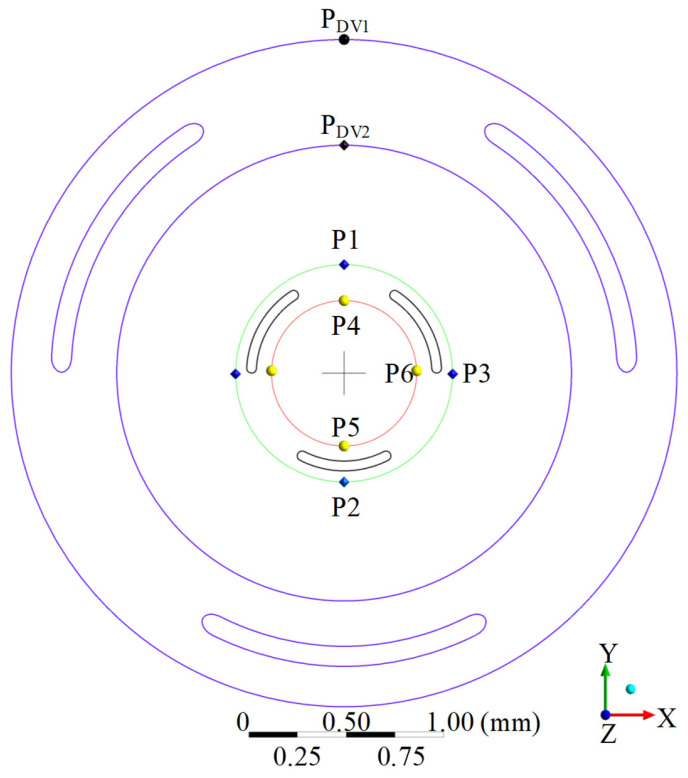
Optimal solution of die land’s inlet and outlet of the extrudate.

Figure 10 shows the inlet cross section of the die land and the coextruded product cross section obtained through the optimized design. The optimal value of the inner diameter of the die runner (DV1) was 3.413 mm, the outer diameter of the tip end was 2.331 mm, and the geometric dimensions of the inlet of the auxiliary material were determined by the numerical simulation.

The average outer diameter of the coextruded product was 1.109 mm (P1 was 1.109 mm, P2 was 1.109 mm, and P3 was 1.108 mm), and the average inner diameter of the extrusion was 0.788 mm (P4 was 0.794 mm, P5 was 0.781 mm, and P6 was 0.789 mm). Since the inlet geometry of the auxiliary material was designed by the inverse extrusion method, the outlet geometry of the free jet of the auxiliary material was consistent with the expected value. The cross-sectional area of the total, main, and auxiliary materials was 0.263 mm^2^, 0.227 mm^2^, and 0.036 mm^2,^ respectively. The expected value of the cross-sectional area of the main body was 0.187 mm^2^, and the expected value of the cross-sectional area of the auxiliary material was 0.0365 mm^2^. The discretization of the finite element caused an error in the cross-sectional area of the auxiliary material.

**Figure 11:**
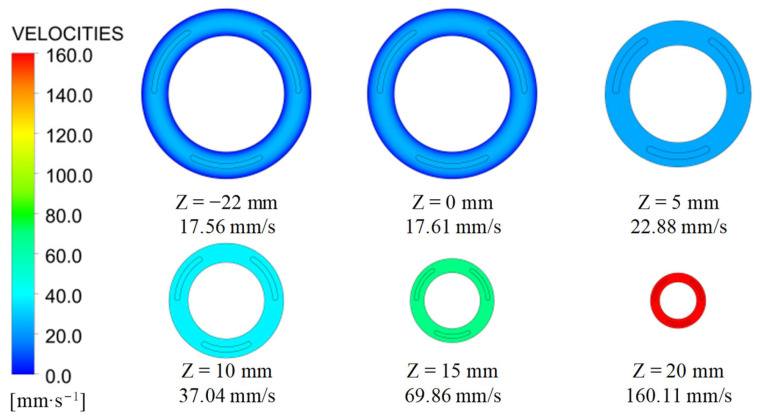
Average velocity in the Z direction.

The velocity distribution along the Z direction during the coextrusion process is shown in Figure 11. The inlet of the extrusion die was Z = −22 mm, the die lip was Z = 0 mm, and the outlet of the extrudate was Z = 20 mm. In the extrusion die, the flow of the polymer melt was stable, and the average velocity at the die lip increased slightly. After the polymer melt left the extrusion die, under the action of the traction device, the flow rate of the polymer melt gradually increased to 160.11 mm/s, and the cross-sectional area of the coextruded product decreased to the target value accordingly.

The average velocity and cross-sectional area of the main material (TPU) and auxiliary material (modified TPU) at different positions in the Z direction are shown in Table 3. As a consequence of take-up [44], the flow velocity of the coextrusion fluid underwent a gradual increase, while the cross-sectional area experienced a corresponding decrease.

Since the main material was in contact with the inner and outer walls of the coextrusion die, the wall friction resistance was relatively large, and the average flow rate in the die was relatively low. The auxiliary material had no contact with the wall of the die. At the same time, it was in the center of the extrusion, and its average flow rate within the die was relatively large. At the extrusion die inlet, the main material’s average velocity was 16.73 mm/s, and that of the auxiliary material was 25.57 mm/s. At the extrusion die’s exit, the main material’s average velocity was 16.88 mm/s, which was 0.9% higher than that at the inlet. At the extrusion die’s exit, the auxiliary material’s average velocity was 24.29 mm/s, which was 5% lower than that at the inflow. The area percentage of the auxiliary material at the exit of the die was 9.84%, slightly larger than that at the entrance. These results suggest a slight interfacial movement within the die [2,15].

Upon the extrusion exiting the die, a distinct phenomenon became evident: the area percentage occupied by the main material gradually decreased, while the area percentage occupied by the auxiliary material concurrently increased. This observation provides compelling evidence of a swelling-like phenomenon [2,15] occurring after the auxiliary material leaves the die.

At the outlet of the extrudate, the average speed of the main and auxiliary materials was 160.11 mm/s. At the extrusion die lip, the velocity distribution of the two materials was non-uniform, but after the die swell, the average velocity was equal at the position of Z = 5 mm until the exit of the extrudate. The inlet design of the main material adopted the optimization design method, and the cross-sectional area of the extruded part met the tolerance requirements. The inlet design of the auxiliary material adopted the inverse design method, and the cross-sectional area of the extruded part was consistent with the expected value.

**Figure 12:**
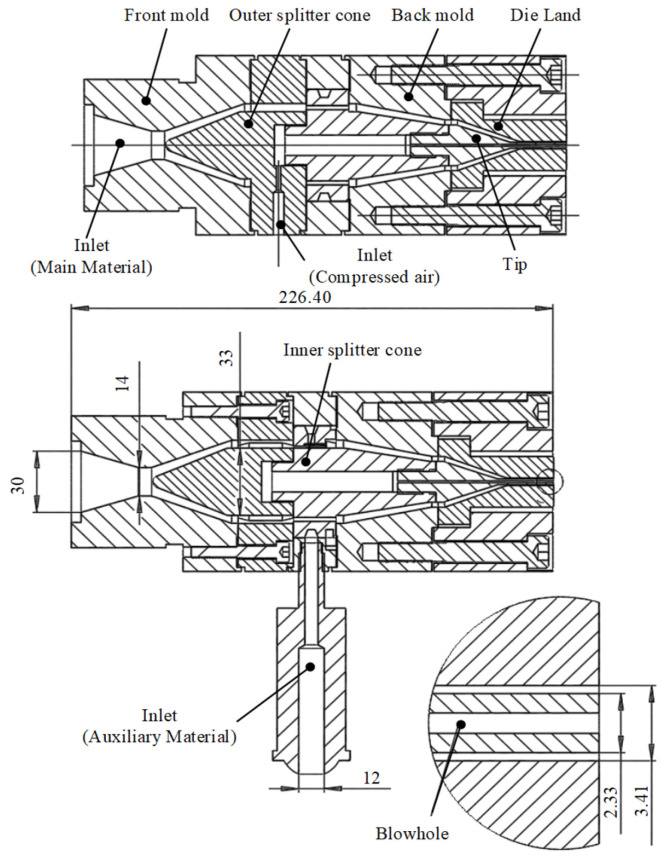
Drawing of coextrusion die.

According to the optimal solution above, a coextrusion die was designed, as shown in Figure 12. The coextrusion die material was S136H steel, and a cross-shaped coextrusion die was used [26]. The main material inlet adopted a straight-through structure, and the inlet flow direction paralleled the outlet flow direction. The auxiliary material inlet adopted a right-angle structure, and the inlet flow direction was perpendicular to the outlet flow direction. The modified TPU melt rotated 90° and then entered the confluence channel. Compressed air also adopted a right-angle structure and entered the die from the splitter cone.

The main material extruder was an SH30 single-screw extruder, the screw diameter was 30 mm, and the aspect ratio was 25:1. The auxiliary material extruder was an SH25 single-screw extruder, and the screw diameter was 25 mm. The aspect ratio was 25:1. Before introducing the materials, a thorough drying process was conducted individually for both the main material and auxiliary materials. They were subjected to baking in a precision warm air oven at 120 °C for a duration of 4 h to achieve a moisture content below 0.02 wt%. The extruder utilized in the study was equipped with an advanced temperature control system, providing a remarkable temperature control accuracy of ±1 °C. This precise temperature control was essential for maintaining optimal processing conditions during the coextrusion process. The heating temperature of the extrusion die was 195 °C, the temperatures of the three heating zones from the barrel inlet to the outlet of the extruder for the main material were 205 °C, 207 °C, and 210 °C, respectively, and the screw speed was 10 r/min. The temperatures of the three heating zones of the auxiliary material extruder were 210 °C, 220 °C, and 230 °C, respectively, and the screw speed was 15 r/min.

**Figure 13:**
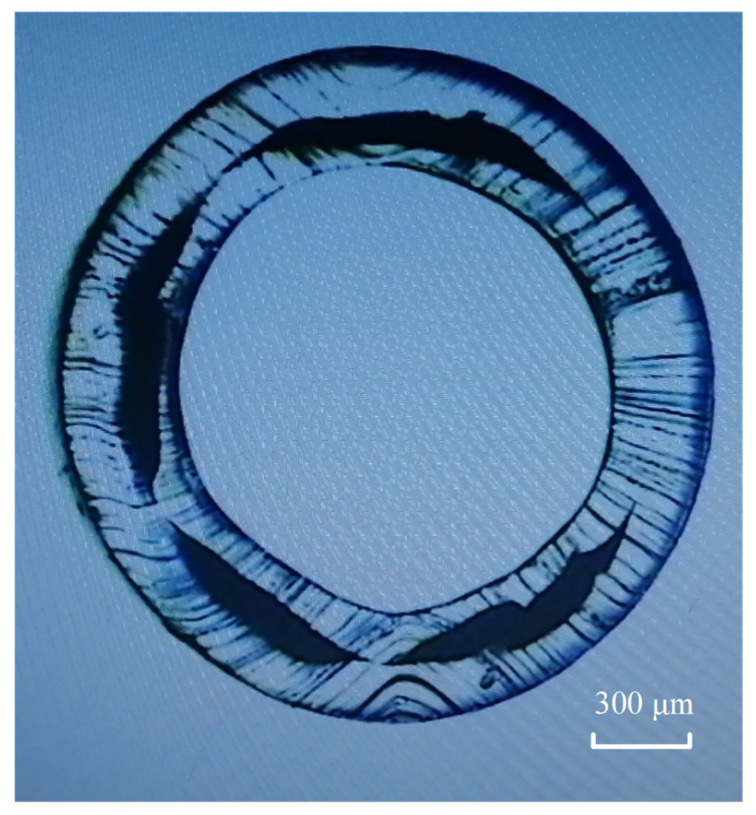
Cross-section image of the catheter made.

An example of the experimental results is shown in Figure 13. The outer and inner shapes of the main material resembled regular circles, but the auxiliary material had interface instabilities. Several factors can influence the material interface stability [15,45], including viscous encapsulation, elastic layer rearrangement, and interfacial flow instabilities. Interfacial instability is shown here as a result of the viscous encapsulation. As depicted in Figure 2, the shear viscosity of the modified TPU was observed to be slightly higher than that of the TPU within the shear rate range of 0.1 s^−1^ to 77 s^−1^. However, when the shear rate exceeded 77 s^−1^, the shear viscosity of the modified TPU became lower than that of the TPU. Notably, during the coextrusion process, the longer die land led to shear rates higher than 77 s^−1^, resulting in the viscosity of the modified TPU being lower than that of the TPU. Consequently, the modified TPU melt tended to encapsulate the TPU melt due to the energetically favored state of minimal pressure loss. To address the aforementioned problems, it was necessary for the viscosity of the TPU melt to be lower than that of the modified TPU melt. One potential solution involved appropriately increasing the temperature of the TPU melt to reduce its viscosity, or conversely, reducing the temperature of the modified TPU melt. This adjustment in temperature aided in achieving the desired viscosity ratio between the two melts, thereby mitigating the interfacial instability and promoting more stable coextrusion processes.

The experimental extrudates were randomly selected, and their outer and inner diameters were measured, as shown in Table 4. The outer diameter was required to be 1.10 ± 0.01 mm, and the inner diameter was expected to be 0.80 ± 0.05 mm. The optimization design results revealed that the average outer diameter of the extrudate measured was 1.10 ± 0.01 mm, while the average inner diameter was 0.79 ± 0.01 mm. Remarkably, the experimental results aligned closely with the optimal solution, and all test samples demonstrated the desired geometric accuracy, fulfilling the design requirements. This shows the effectiveness of the optimization design method in determining the optimal geometric parameters of the coextrusion die’s flow channel. Consequently, this approach improved the precise production of the extruded products [21], and significantly reduced the design cycle duration and the costs. The successful implementation of the optimization design method not only streamlined the extrusion die design process but also enhanced the overall efficiency and quality of the extrusion process.

## 7. Conclusions

This study proposed a novel coupling method for a coextrusion die design to solve the challenges due to the die swell and interfacial movement in the polymer coextrusion process. The method took advantage of the high precision capabilities of the optimization design method and the high efficiency of the inverse design method, which significantly improved the coextrusion die design process and provided better performance and productivity. The coupling method developed will provide a practical and an effective solution to the industry.

(1) A three-dimensional finite element method was utilized to analyze comprehensively the coextrusion flow of TPU and the modified TPU in the die–extrudate region. The results of the analysis clearly demonstrated that the geometric parameters played a crucial role in determining the overall design of the coextrusion die.
(2) An improved optimization design approach for coextrusion dies was developed to achieve the desired geometric accuracy for the extrudate. Through the implementation of a global optimization design, a significant reduction in the objective function was achieved. The value of the objective function was reduced by 72.3%, decreasing from an initial value of 1036.5 to a final value of 287.35.
(3) The improved accuracy was achieved by the global optimization process through the implementation of the local inverse design method. An analysis of the results revealed that the moving interface exhibited a swelling phenomenon upon exiting the die. Consequently, it was necessary to reduce the cross-sectional entrance area of the modified TPU to compensate for this effect. The use of the inverse design methodology proved to be a highly efficient approach in accomplishing this task without the need for intricate optimization designs. Furthermore, the inverse design effectively avoided the mutual coupling between the die swell and the interface, thereby ensuring a more streamlined and efficient design process.
(4) The experimental results demonstrated that the catheter exhibited a high level of geometric accuracy. The experimental results revealed that the average outer diameter of the extrudate measured was 1.10 ± 0.01 mm, while the average inner diameter was 0.79 ± 0.01 mm. These measurements fell well within the acceptable range of error. However, it is important to acknowledge that interface instability arose at the coextrusion interface due to the influence of viscous encapsulation. The underlying causes for this instability necessitate further experimental investigations and verifications in future studies.

## Figures and Tables

**Table 1:** Parameters of the Bird–Carreau model.

Material	Zero Shear Viscosity	Infinite Shear Viscosity	Natural Time	Non-Newtonian Index
	$\eta_{0}\;(\mathrm{Pa}\cdot s)$	$\eta_{\infty }\;(\mathrm{Pa}\cdot s)$	λ (s)	n
TPU	339.637	14.8966	18.2622	0.493359
TPU with 30 wt% BaSO_4_	2801.85	4.34606	3253.47	0.592924

**Table 2:** Design variables and value ranges (Unit: mm).

No.	Description	Initial Value	Value Ranges
DV1	Inner diameter of die runner	3.3	3.25 ≤ DV1 ≤ 3.45
DV2	Outer diameter of tip end	2.4	2.2 ≤ DV2 ≤ 2.6

**Table 3:** Average velocity in Z direction and cross-sectional area of main and auxiliary materials.

Z Coordinate	Total		Main Material		Auxiliary Material	
	Average Velocity (mm/s)	Area (mm^2^)	Average Velocity (mm/s)	Area (%)	Average Velocity (mm/s)	Area (%)
−22	17.56	2.439	16.73	90.65%	25.57	9.35%
0	17.61	2.439	16.88	90.16%	24.29	9.84%
5	22.88	1.880	22.88	86.44%	22.88	13.62%
10	37.04	1.158	37.04	86.36%	37.04	13.64%
15	69.86	0.619	69.86	86.43%	69.86	13.60%

**Table 4:** Measured values of the outer and inner diameters of the catheter (Unit: mm).

No.	Outer	Inner	No.	Outer	Inner	No.	Outer	Inner
1	1.109	0.78	11	1.095	0.79	21	1.091	0.79
2	1.097	0.78	12	1.101	0.80	22	1.106	0.80
3	1.099	0.79	13	1.105	0.79	23	1.109	0.79
4	1.097	0.78	14	1.094	0.79	24	1.098	0.79
5	1.094	0.79	15	1.099	0.81	25	1.095	0.81
6	1.110	0.80	16	1.095	0.78	26	1.106	0.78
7	1.108	0.78	17	1.110	0.78	27	1.110	0.78
8	1.091	0.79	18	1.097	0.79	28	1.097	0.79
9	1.093	0.80	19	1.096	0.82	29	1.105	0.82
10	1.094	0.81	20	1.107	0.79	30	1.099	0.79

## Data Availability

The data presented in this study are available on request from the corresponding author. The data are not publicly available due to privacy.
